# Stress in farmed saltwater crocodiles (*Crocodylus porosus*): no difference between individually- and communally-housed animals

**DOI:** 10.1186/2193-1801-2-381

**Published:** 2013-08-13

**Authors:** Sally R Isberg, Cathy M Shilton

**Affiliations:** Centre for Crocodile Research, PO Box 329, Noonamah, Northern Territory 0837 Australia; Faculty of Veterinary Science, University of Sydney, Sydney, NSW 2006 Australia; Berrimah Veterinary Laboratories, Department of Primary Industry and Fisheries, Northern Territory Government, Berrimah, Northern Territory 0800 Australia

**Keywords:** Communal pens, Corticosterone, Individual pens, Saltwater crocodile, Stress

## Abstract

Minimising stress in farmed crocodiles is not only important for improving animal welfare, but may also improve skin blemish healing and infection resistance, which influence the quality of the final skin product. Forty near-harvest size saltwater crocodiles (1.6-1.8 m TL) from two Australian farms were sampled to evaluate the effect of different pen types (communal pens n=20; individual pens n=20) on stress as indicated by plasma corticosterone. Blood samples were taken within three minutes of immobilisation and analysed using a commercial enzyme immunoassay kit. There was no relationship with animal size (*P*=0.16), between farms (*P*=0.86), pen types (*P*=0.69), communal pens between farms (*P*=0.28) or individual pens between farms (*P*=0.24). Based on corticosterone levels, it appears that individual pens do not cause significantly more stress on harvest-size animals than communal pens. Individual pens meet their design specifications by achieving comparable healing rates of belly skin blemishes as communal pens without compromising animal welfare and minimising the possibility of new blemishes.

## Introduction

Quality specifications of raw crocodile skins imposed by skin buyers have become increasingly important over recent years as the worlds’ production of crocodilian skins increases. Although saltwater crocodile (*Crocodylus porosus*) belly skin remains in high demand due to its desirable traits of evenly distributed small scales, the definition of a “blemish-free” skin (Isberg et al. 2004) is becoming increasingly rigorous. However, publications detailing the pathological and epidemiological aspects of pre-harvest crocodilian skin blemishes are scarce. Anecdotal evidence suggests that many belly skin blemishes are superficial scratches and punctures that have been caused by non-aggressive interactions between conspecifics within communal pens (Huchzermeyer 2003). The majority of these scratches and punctures only penetrate the upper keratin (scale) layer, whilst some go slightly deeper into the underlying epidermal and dermal layers. Few penetrate through the skin into the underlying musculature, although aggressive interactions can cause these more severe wounds. Other factors such as rough concrete can also cause skin damage (Huchzermeyer 2003).

Experience with *C. porosus* has shown that the majority of superficial blemishes on the belly skin heal given adequate time. As a result, many saltwater crocodile skin producers are now using individual pens as a finishing production stage. These individual pens allow blemishes to heal without the risk of more being added by conspecifics. Although juvenile and adult saltwater crocodiles are largely solitary and intolerant of conspecifics in the wild (Webb and Messel 1977,1978), on farms they are reared in groups from hatching until finishing. It is therefore possible that being placed in a solitary situation at the finishing stage may increase stress.

Prolonged stress and resultant chronic exposure to glucocorticoid stress hormones in mammals is associated with a myriad of negative health effects, including decreased infection resistance and altered wound healing (Schobitz et al. 1994; Christian et al. 2006; Capen 2007; Marketon and Glaser 2008; Poetker and Reh 2010). Crocodilians have a similar hypothalamic-pituitary-adrenal system and secrete the glucocorticoid hormone corticosterone in response to stress (Lance et al. 2000). There is some evidence that stress causes immunosuppression and altered wound healing in crocodilians and lizards (Morici et al. 1997; Lance et al. 2000; French et al. 2006). Wound infection and slow or altered healing dynamics in farmed crocodiles near the finishing stages can negatively influence the quality of the final skin product. The purpose of this study was to investigate if housing harvest-size saltwater crocodiles individually increases stress, as indicated by corticosterone, compared to communal housing.

## Methods and materials

### Experimental housing

This study was conducted on two crocodile farms in the Northern Territory, Australia. On both farms, saltwater crocodiles were raised in communal pens from hatching until slaughter size (1.6 – 1.8 m), and thereafter in individual pens for finishing. The communal pens on both farms were similar in design (concrete floors (60:40 land:water ratio), corrugated iron walls and canvas roof covering), with a north–south orientation and the same stocking density (0.57 m^2^/animal). However, the communal pens on Farm 1 (CP1) have only one body of water whereas the grow-out pens on Farm 2 (CP2) have two bodies of water. The individual pens on both farms (Farm 1: IP1; Farm 2: IP2) were also similar in design constructed of concrete blocks with a 70:30 land:water ratio and allow 1.22 m^2^/animal. All animals were fed chicken heads in excess in the late afternoon/early evening with uneaten food removed the following morning, and the pens cleaned with a sodium hypochloride solution (Chlorfoam, Reward Distribution, Darwin) and the water changed. Crocodiles were fed either 2 (individual pens) or 3 (communal pens) times a week during the wet (hot, humid) season and this was reduced to 1 (individual pens) or 2 (communal pens) times a week during the dry (cool, dry) season as food consumption declined.

### Experimental animals

From each farm, ten crocodiles from individual pens (IP1: 10; IP2: 10) and ten from communal pens (CP1: 10; CP2: 10) were sampled (total of 40). Blood (2-3 ml) was taken from the occipital sinus as described by Lloyd and Morris (1999) using 18 gauge 1” needles into serum vacutainers within three minutes of electrical immobilisation as described by Franklin et al. (2003) to ensure basal corticosterone levels were obtained. From the communal pens, five animals were sampled within ten minutes from two different pens to minimise the possible effect of prolonged human presence on basal corticosterone levels. This also provided a more representative sample of animals in communal pens. Total length (TL) was measured on each crocodile from the tip of the snout to the tip of the tail to investigate any effect of size on serum corticosterone (Table 1).

**Table 1:** **Average (standard error of the mean; SEM) total length and corticosterone (ng/ml) in the communal (CP) and individual pens** (IP) **at Farms 1 and 2**

	Pen type	N	Total length (cm)	Corticosterone (ng/ml)
Farm 1	CP1	10	158.0 (2.3)	8.69 (5.53)
	IP1	10	159.4 (3.3)	3.17 (1.44)
Farm 2	CP2	10	165.3 (3.2)	3.04 (0.96)
	IP2	10	174.8 (2.4)**	6.34 (2.05)
**Overall average**		**40**	**164.4 (1.7)**	**5.31 (1.52)**

N is the number of animals in each group.

**indicates significantly difference (*P*<0.01) in animal size.

### Corticosterone assay

Serum corticosterone was measured using the OCTEIA Corticosterone HS enzyme immunoassay kit (IDS Ltd., Tyne & Wear, UK) as per kit instructions. The limits of accurate detection for the kit, according to the kit specifications, are 0.17-15 ng/ml. As such, sample results less than 0.17 ng/ml were set to 0, and to accurately interpret samples that may have exceeded the 15 ng/ml upper limit, testing was performed on neat and diluted (either 1:2 or 1:10) aliquots. An appropriate model for conversion of percentage binding values to corticosterone in ng/ml was determined using the kit calibrator values. CurveExpert (2009, version 1.4) curve fitting software was used to model the calibration curve with best-fit curves determined by their standard error and correlation coefficients. The correlation of the undiluted and the diluted serum corticosterone was 0.94 showing the validity of using the kit in this manner.

### Statistical analysis

The data were log-transformed and analysed using Generalised Linear Models (GLM) and analysis of variance in Genstat (2011, version 14) using variations of the following model.1

where LnCort_*ijkl*_ is the natural logarithmic transformation of corticosterone (ng/ml); μ is the overall mean; TL_*i*_ = total length (TL) of the *i*th individual; β_TL_ = regression coefficient for TL; Pen type_*j*_ is the fixed effect of either communal or individual pen on the respective farm (j = CP1, CP2, IP1, IP2); Farm_*k*_ is the fixed effect of the *k*th farm (k = 1,2); SamplingOrder_*il*_ is the effect of the sampling order (l = 1,..,5) on the *i*th individual; and ϵ_*ijkl*_ is the random error [assumed N(0,)].

## Results

### Animal size versus corticosterone value

**Figure 1:**
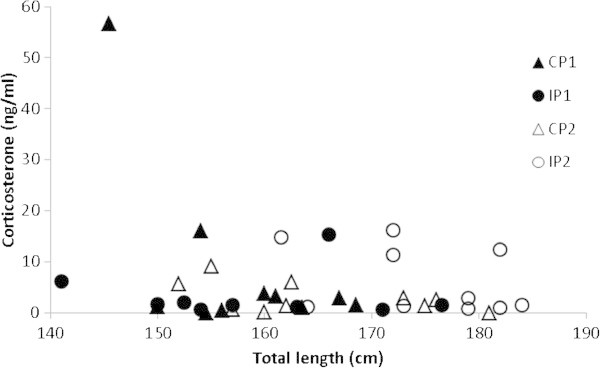
**Relationship between animal size (total length; TL) and corticosterone value (ng/ml) according to pen type: communal (Farm 1 – solid triangles; Farm2 – open triangles) and individual pens (Farm 1 – solid circles; Farm 2 – open circles) (*P*=0.16).**

The overall average corticosterone value for all crocodiles on both farms was 5.31 ± (SEM) 1.52 ng/ml. The individual pen animals from Farm 2 (IP2) were significant larger than the other crocodiles (P<0.01; Table 1). Irrespective, as Figure 1 shows, there is no relationship between animal size (total length; TL) and corticosterone value (*P*=0.16).

### Farm 1 versus farm 2

To evaluate any significant farm effects on the animals, a basic analysis of Farm 1 versus Farm 2 including all animals irrespective of pen type was conducted. There were no significant differences between farms (*P*=0.86).

### Communal versus individual pens

After establishing there was no difference between farms, an overall analysis was run to evaluate if there were any differences between communal and individual pens. No relationship was found (*P*=0.69).

### Pen type between farms

**Figure 2:**
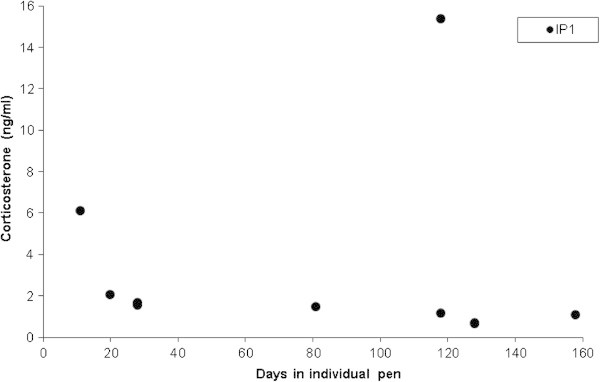
**Corticosterone levels (ng/ml) of crocodiles in individual pens from Farm 1 against the number of days in the pen (*P*=0.83).**

By sub-setting the data into communal and individual pen types, the overall average corticosterone value of communal pen animals was 5.86 ± 2.81 ng/ml (range 0–56.67 ng/ml) whilst the average corticosterone value of individual pen animals was 4.75 ± 1.27 ng/ml (range 0.66-16.16 ng/ml). An analysis comparing communal and individual pens between Farms 1 and 2 showed there were no significant differences between farms (*P*=0.28 and *P*=0.24, respectively). Records on duration in individual pens were available for Farm 1. There was no relationship between how long an animal had been in an individual pen and their corticosterone value (*P*=0.83, Figure 2).

### Sampling order

**Figure 3:**
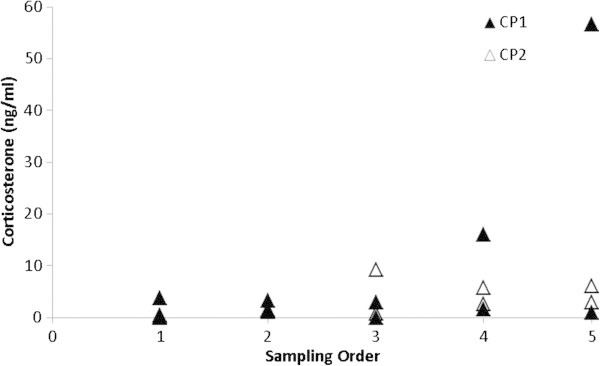
**The order crocodiles were blood sampled (Sampling Order) from communal pens for corticosterone assay (ng/ml).** Communal animals from Farm 1 (CP1; solid triangles) and Farm 2 (CP2; open triangles) (*P*=0.08).

The order each animal was sampled within communal pens did not significantly effect corticosterone levels (*P*=0.08). However, there was a trend towards increased corticosterone with prolonged human presence in the pen (Figure 3).

## Discussion

Previous studies of stress in crocodilians have shown a negative relationship between corticosterone and immune function (Morici et al. 1997), juvenile growth rates (Elsey et al. 1990; Morici et al. 1997; Turton et al. 1997), juvenile mortality (Morici et al. 1997), the major reproductive hormones (testosterone – Lance and Elsey 1986; estradiol – Elsey et al. 1991) and reproductive success (Lance 1994). Plasma corticosterone has also been used to quantify crocodilian stress imposed by handling (Gist and Kaplan 1976; Lance and Elsey 1999), different restraint methods (manual versus immobilisation; Franklin et al. 2003), different stocking densities (Elsey et al. 1990), environmental salinity (Lauren 1985; Lance et al. 2010) and between healthy and runt crocodiles of similar age (Isberg et al. 2009). In this study, no significant differences were found in corticosterone levels, and thus stress, between harvest-size saltwater crocodiles (1.6-1.8 m TL) housed in communal or individual pens.

The only other study that has examined corticosterone levels in saltwater crocodiles of a similar size to this study and in a farm situation was Franklin et al. (2003), whilst investigating the the effect of immobilisation compared to manual restraint. Franklin et al. (2003) reported baseline corticosterone values of 1.09 ± 0.28 and 1.08 ± 0.14 ng/ml for the immobilised and manual restraint groups respectively, with a maximum average corticosterone value of 2.25 ng/ml half an hour following manual restraint. All of the animals sampled in Franklin et al. (2003) were housed in individual pens for three months prior to sampling. If only the individual pen animals from this study are considered, the results presented herein show higher overall corticosterone levels (average 5.31 ± 1.52 ng/ml) compared to Franklin et al. (2003). The reason for this discrepancy is unclear. However, the ambient air temperatures (average 22.9°C; range 14-32°C) in Franklin et al. (2003) were much lower than in the current study (33.2°C and 32.9°C for Farms 1 and 2, respectively; http://www.bom.gov.au). Further work is underway to establish a relationship between temperature/seasonal effects on crocodile corticosterone levels.

There was no statistically significant relationship between length of time in individual pens and corticosterone level on Farm 1 (Figure 2). However, if there were more data available, it is conceivable that Figure 2 could represent the return to baseline corticosterone values after moving from a communal to individual pen. That is, one animal sampled 11 days after being placed in an individual pen had a corticosterone value of 6.11 ng/ml, another after 20 days in an individual pen had a value of 2.04 ng/ml and then values stabilise around 1.17 ± 0.15 ng/ml (n = 7; 28–158 days in individual pens). The one exception from Farm 1 was an animal that had been in its individual pen for 118 days (corticosterone value of 15.35 ng/ml).

The remainder of the literature on corticosterone levels in crocodilians concerns either alligators (*Alligator mississippiensis*) and/or animals of a different age than those used in this study. In a study involving corticosterone implants in alligators less than 12 months old, the placebo (control) group showed high variation in serum corticosterone, ranging between 3.8-42.8 ng/ml (Morici et al. 1997). A similar range of corticosterone values have been reported in adult alligators at different stocking densities (Elsey et al. 1990) and alligators (<12 months old) subject to restraint (Lance and Elsey 1999). In sexually mature adult alligators, published baseline corticosterone values range from 0.07–1.86 ng/ml in females (Elsey et al. 1991) and from 0.27-1.99 ng/ml in males (Lance and Elsey 1986). In saltwater crocodiles, Turton et al. (1997) reported average corticosterone levels of 6.82 ± 0.3 ng/ml, with a range between 0.24 and 15.29 ng/ml, for less than 14 week old saltwater crocodile hatchlings. In 5–7 month old saltwater crocodiles, corticosterone averaged 10.13 ng/ml in normal crocodiles and 16.18 ng/ml in animals that were small for their age (Isberg et al. 2009). This large variation in serum corticosterone presented in the published literature and reflected in the present study indicates there is still considerable work to be done to understand the underlying dynamics of crocodilian corticosterone secretion.

Corticosterone levels of saltwater crocodiles in this study are generally comparable to published values for this species and alligators in captivity. Our results suggest there is no difference in corticosterone levels between saltwater crocodiles housed communally or individually. As maintaining low stress levels may be important for general well-being, prevention of infections and healing of existing skin blemishes in crocodiles, housing saltwater crocodiles of this size in individual pens is not considered detrimental. Individual housing also has the advantage of preventing new skin blemishes caused by conspecifics from occurring.
